# Around the Hospitals

**Published:** 1893-12-23

**Authors:** 


### Around the Hospitals.

Notice to the Managers of Institutions.—Special space is now reserved for the insertion of notices of hospital meetings and festivals. The Editor requests that all notices may reach The Hospital Office, 428, Strand, at least one week before the date of each meeting.

**The Glasgow Western Infirmary.**—The Western Infirmary shows two notably good things in its report for the past year. These are an increase of patients treated and a decrease of expenditure. The number of in-patients exceeded that of the previous year by 186, whilst the expenditure shows a diminution of no less than £758 The ordinary reliable income of the infirmary falls far short of the expenditure, but hitherto it has been very fortunate in the receipt of donations and bequests, which have supplied the required funds. Annual subscriptions show a slight increase, but the ordinary income from all sources is less, a fact accounted for by disturbances in trade, and from which the management congratulate themselves they have not suffered more severely. Various additions to the institution are becoming urgently needed. A new dispensary, operating rooms, and a pathological building are required for the medical department, whilst in the household the wash-house and laundry require enlarging and new appliances. The pathological building and laundry extensions are already in progress. To render the infirmary complete £40,000 will be required, and of this the management have but £10,000, a grant from the Bellahouston Trust. They therefore rely on the public for assistance. As the sphere of the infirmary extends a larger amount of help will be required. The Lady Hozier Convalescent Home, opened in July, and which is affording such material assistance to the hospital, entails an extra expenditure of about £1,500 per annum. No one could wish one iota of the useful work of the Western Infirmary curtailed, and therefore it is to be hoped that a cheerful response to its appeals will be made.

**The North Staffordshire Infirmary.**—The past year has presented no particular features of note except the unusual number of typhoid patients treated, so much so that the fever wards of the institution were utilised. Here as in many other establishments the depression in trade has been felt, but unusually large sums having come in from legacies the difficulty has been tided over, and the income for the year amounted to £11,754, as against £11,622 for the year preceding. The expenditure has been unusually large, owing to the adoption of new heating arrangements, whereby the wards can be kept at a sufficiently high temperature for medical cases, which was not the case previously. At the annual meeting a rule was passed prohibiting the reception of patients suffering from Asiatic cholera.

**The Rotunda Lying-in Hospital, Dublin.**—It is the practice of the Hospital Sunday Fund in Dublin to inspect all hospitals participating in their distribution of grants. The last report of this Visiting Committee shows improvements have taken place in most of the directions considered desirable, and the amounts received from various other sources prove that the hospital has the public confidence. The number of patients continue to increase, and alterations and additions will be made as soon as funds are forthcoming; £6,273 has already been received towards the £10,000 necessary to complete the whole. The charity is a most useful one, and it is desirable that it should be placed on the best footing. Many societies and private persons occupy themselves in rendering assistance to the institution in various directions. The cab fund, by means of which patients are conveyed to the hospital when necessary, fulfils a most useful office.

